# Mutation analysis of *CRYAA*, *CRYGC*, and *CRYGD* associated with autosomal dominant congenital cataract in Brazilian families

**Published:** 2009-04-17

**Authors:** Alessandro Santana, Mauro Waiswol, Enyr Saran Arcieri, José Paulo Cabral de Vasconcellos, Mônica Barbosa de Melo

**Affiliations:** 1 Cataract Service, Department of Ophthalmology, Faculty of Medical Sciences, Irmandade da Santa Casa de Misericórdia de São Paulo, São Paulo, Brazil; 2 Glaucoma Service, Department of Ophthalmology, Faculty of Medical Sciences, University of Campinas - UNICAMP, Campinas, São Paulo, Brazil; 3 Laboratory of Molecular Medicine, Department of Physiology, Faculty of Medical Sciences, Irmandade da Santa Casa de Misericórdia de São Paulo, São Paulo, Brazil; 4 Laboratory of Human Molecular Genetics, Center for Molecular Biology and Genetic Engineering (CBMEG), University of Campinas – UNICAMP, Campinas, São Paulo, Brazil

## Abstract

**Purpose:**

Congenital cataracts are one of the most treatable causes of visual impairment and blindness during infancy. Approximately 50% of all congenital cataract cases may have a genetic cause. Once there is an intimate relationship between crystallin genes and lens transparency, they are excellent candidate genes for inherited cataract. The purpose of this study was to investigate mutations in αA-crystallin (*CRYAA*), γC-crystallin (*CRYGC*), and γD-crystallin (*CRYGD*) in Brazilian families with nuclear and lamellar autosomal dominant congenital cataract.

**Methods:**

Eleven Brazilian families were referred to the Santa Casa de São Paulo Ophthalmology Department. The coding regions and intron/exon boundaries of *CRYAA*, *CRYGC*, and *CRYGD* were amplified by polymerase chain reaction (PCR) and directly sequenced. Mutation screening was performed in the control group by restriction digestion.

**Results:**

Two mutations were observed in different families (Family 4 and Family 10), one is a new mutation (Y56X) in *CRYGD* and the other a previously reported mutation (R12C) in *CRYAA* that is correlated with a different phenotype. Genetic analysis revealed previously described polymorphisms in *CRYAA* (D2D) and *CRYGD* (Y17Y and R95R). A new polymorphism in *CRYGC* (S119S) was identified only in Family 1. The mutations as well as the new polymorphism were not observed in the control group.

**Conclusions:**

In conclusion, we report a novel nonsense mutation (Y56X) in *CRYGD* and a previously reported missense mutation (R12C) in *CRYAA* associated with nuclear cataract in Brazilian families. Both tyrosine in amino acid 56 in *CRYGD* and arginine in amino acid 12 in *CRYAA* have been highly conserved throughout evolution in different species. A new polymorphism (S119S) in *CRYGC* was also observed in one family. The analysis of nine families excluded possible mutations in the crystallin genes, suggesting that other genes could be involved with congenital cataract.

## Introduction

Congenital cataract is the leading cause of reversible blindness in childhood. Its occurrence, depending on the regional socioeconomic development, is 1−6 cases per 10,000 live births in industrialized countries [1,2] and 5−15 per 10,000 in the poorest areas of the world [3,4]. Congenital cataract is visible at birth or during the first decade of life [5]. About 20,000−40,000 new cases of bilateral congenital cataract are diagnosed each year [3,6]. In Brazil, congenital cataract accounts for 12.8% of cases of blindness in childhood [7] due to different causes including metabolic disorders (galactosemia) [8], infections during embryogenesis [1], gene defects, and chromosomal abnormalities [9,10]. Cataract may be an isolated anomaly or part of a multisystem syndrome such as Down syndrome, Wilson’s disease, and myotonic dystrophy [11]. Inherited cataracts correspond to 8%−25% of congenital cataracts [12]. Although X-linked and autosomal recessive transmission has been observed, the most frequent mode of inheritance is autosomal dominant with a high degree of penetrance [13,14]. Inherited cataracts are clinically highly heterogeneous and show considerable interfamilial and intrafamilial variability [15].

Congenital cataracts are also genetically heterogeneous [14]. It is known that different mutations in the same gene can cause similar cataract patterns while the highly variable morphologies of cataracts within some families suggest that the same mutation in a single gene can lead to different phenotypes [15,16]. To date, more than 25 loci and genes on different chromosomes have been associated with congenital cataract [17]. Mutations in distinct genes that encode the main cytoplasmic proteins of human lens have been associated with cataracts of various morphologies [18] including genes encoding crystallins (*CRYA*, *CRYB*, and *CRYG*) [19], lens specific connexins (*Cx43*, *Cx46*, and *Cx50*) [20,21], aquaporin (*MIP*) [22], cytoskeletal structural proteins (beaded filament structural protein 2 [*BFSP2*]) [23], paired-like homeodomain 3 (*PITX3*) [24], avian musculoaponeurotic fibrosarcoma (*MAF*) [25], and heat shock transcription factor 4 gene (*HSF4*) [26]. Crystallin proteins (α-, β-, and γ-crystallins) represent more than 90% of lens soluble proteins in humans, encompassing almost 35% of its mass and accounting for its optical transparency and high refractive index [6,13].

Mutations in the crystallin genes represent a large proportion of the mutations identified to date. These genes may be divided into two distinct evolutionary groups comprising α-crystallins, which are members of the small heat shock family of molecular chaperones [27], and β/γ-crystallins, which share a common two domain structure composed of Greek-key motifs and belong to the family of epidermis specific differentiation proteins [28].

Lamellar and nuclear congenital cataracts are the most common types of hereditary congenital cataract [29,30]. Several genes have been associated with the presence of these two types of cataracts (*CRYAA*, *CRYBA3/A1*, *CRYGC*, *CRYGD*, *CRYGS*, connexin 46 [*GJA3*], and connexin 50 [*GJA8*]) [13,18,31]. In this study, *CRYAA*, *CRYGC*, and *CRYGD* were considered as candidates for congenital cataract on the basis of both their high levels of expression in the lens and their known function in maintaining lens transparency. Eleven Brazilian families with autosomal dominant nuclear or lamellar congenital cataracts were screened for mutations in *CRYAA*, *CRYGC*, and *CRYGD*.

## Methods

The study protocols adhered to the tenets of the Declaration of Helsinki and were approved by the Human Research Ethics Committees of the Santa Casa de São Paulo School of Medicine (São Paulo, Brazil).

### Patients and control group

Families with a positive history of autosomal dominant bilateral nuclear or lamellar cataract were investigated at Santa Casa de São Paulo Ophthalmology Department. Both affected and unaffected individuals underwent detailed ophthalmic examination including Snellen visual acuity and corrected visual acuity in addition to slit-lamp and fundus examination with dilated pupil, intraocular pressure measurement by applanation tonometry, and corneal diameter. Detailed ocular, medical, and family histories were obtained from each available family member. Probands with a history suggestive of an intrauterine infection such as rubella, unilateral cataract, and other ocular or systemic disorders were excluded from the study. There was no evidence of systemic abnormalities associated with congenital cataract in the probands. After informed consent was obtained from all participating individuals, 5–10 ml of venous blood was collected for genomic DNA extraction and subsequent molecular genetic analysis. Possible mutations and new polymorphisms were analyzed by restriction enzyme digestion in the control group, which was comprised by 50 ophthalmologically normal, unrelated Brazilian individuals from Santa Casa de São Paulo Ophthalmology Department with the same ethnic background and from the same geographic region of the affected group.

### Polymerase chain reaction and DNA sequencing

Polymerase chain reaction (PCR) was used to amplify all the exons and intron/exon boundaries of the candidate genes (*CRYAA*, *CRYGC*, and *CRYGD*). Conditions are described as follows: a 25 μl reaction volume included approximately 100 ng of genomic DNA, 20 pmol of each primer, 1X enzyme buffer (10X buffer=20mM Tris-HCl [pH 8,4] 50 mM KCl, 0,01% gelatin), 1.5 mM MgCl_2_, 200 μM nucleotides (dATP, dCTP, dTTP, dGTP), 0.5 U of Taq DNA polymerase (Invitrogen™ Life Technologies, Carlsbad, CA), and sterile deionized water. Samples were amplified on the MasterCycler EP Gradient S thermal cycler (Eppendorf, Hamburg, Germany) according to the following conditions: initial denaturation at 94 °C for 5 min followed by 35 cycles of 94 °C for 1 min, X °C for 1 min, 72 °C for 1 min, and final extension at 72 °C for 7 min. Oligonucleotide primer pairs, PCR product sizes, and annealing temperatures are described in Table 1. Only the proband of each family has been screened for mutations in the candidate genes.

**Table 1:** Oligonucleotides used as primers for PCR amplification of the *CRYAA*, *CRYGC*, and *CRYGD*, product sizes, and annealing temperatures.

**Gene**	**Exon**	**Strand**	**Sequence (5′→3′)**	**Product Size (bp)**	**T (°C)**
*CRYAA*	1	Sense	CACGCCTTTCCAGAGAAATC	466	59
		Antisense	CTCTGCAAGGGGATGAAGTG		
	2	Sense	CTTGGTGTGTGGGAGAAGAGG	377	57
		Antisense	TCCCTCTCCCAGGGTTGAAG		
	3	Sense	CCCCCTTCTGCAGTCAGT	989	66
		Antisense	GCTTGAGCTCAGGAGAAGGA		
*CRYGC*	1–2	Sense	ACCAGAGAACAAGGACACAATC	674	62
		Antisense	TGGCTTATTCAGGTCTCTGATG		
	3	Sense	ATTCCATGCCACAACCTACC	590	62
		Antisense	CCAACGTCTGAGGCTTGTTC		
*CRYGD*	1–2	Sense	CCCTTTTGTGCGGTTCTTG	596	58
		Antisense	TTTGTCCACTCTCAGTTATTGTGAC		
	3	Sense	TGTGCTCGGTAATGAGGAG	700	62
		Antisense	AGGCCAGAGAATCAAATGAG		

PCR products were electrophoresed in 1.5% agarose gels containing 0.05% ethidium bromide, purified, and submitted to direct sequencing on the ABI PRISM 310 Genetic Analyzer automated sequencer (Applied Biosystems, Foster City, CA). The sequencing reactions were performed under the following conditions: 25 cycles of 96 °C for 10 s, 56 °C for 5 s, and 60 °C for 4 min, using Big Dye Terminator Ready Reaction v3.1 (ABI PRISM Big Dye Terminator Cycle Sequencing Kit; Applied Biosystems). Sequencing results were analyzed through submission to similarity search using the “search algorithm” BLAST. Any sequence variation suggestive of a mutation was later confirmed in parents and available relatives to determine the segregation with the disease.

### Restriction digestion

Genetic variations were evaluated in the control group by the presence/absence of cleavage sites for the restriction enzymes HhaI for *CRYAA*, MslI for *CRYGC*, and RsaI for *CRYGD* (New England BioLabs, Ipswith, MA). Digestions were conducted in accordance with the manufacturer’s protocols. The resulting restriction fragments were separated in 2% agarose gels stained with ethidium bromide and analyzed under ultraviolet (UV) light.

### Computational methods

Besides the analysis of the control group, computational algorithms were used to predict which variants were deleterious and which were neutral. Three methods were used to determine if a specific amino acid substitution within a protein sequence might lead to altered protein function and possibly contribute to the disease. Automated methods available on the Internet were used: Sorting Intolerant from Tolerant amino acid substitutions (SIFT), Polymorphism Phenotyping (PolyPhen), and Grantham score difference (Align-GVGD).

SIFT uses an evolutionary approach and is based on the assumption that important amino acids tend to be conserved across species. SIFT assigns a substitution probability from 0 to 1 for each possible amino acid change. Substitution with probabilities less than 0.05 are considered intolerant (i.e., functionally significant) whereas those greater than or equal to 0.05 are inferred as tolerated substitutions. PolyPhen takes into account not only the evolutionary conservation of the amino acid subjected to the mutation but also the physico-chemical characteristics of the wild type and mutated amino acid residue and the consequence of the amino acid change for the structural properties of the protein. Align-GVGD is based on chemical differences between each amino acid pair, polarity, and molecular volume. The matrix of scores varies from a minimum of 15 to a maximum of 215. Deleterious mutations tend to have scores greater than 100 and tolerated variations scores less than 60 [32,33].

## Results

Eleven families with autosomal dominant childhood cataracts were identified, seven families presenting with the nuclear phenotype and the remaining families with the lamellar phenotype (Table 2). A total of 34 affected members and 44 unaffected members were evaluated in this study.

**Table 2:** Cataract phenotypes, mutations, and polymorphisms identified in this study.

** Family ID**	** Cataract phenotype**	**Mutation**			**Polymorphism**		
		***CRYAA***	***CRYGC***	***CRYGD***	***CRYAA***	***CRYGC***	***CRYGD***
Family 1	Nuclear+Microcornea				D2D	S119S	Y17Y, R95R
Family 2	Lamellar				D2D		R95R
Family 3	Nuclear				D2D		Y17Y, R95R
Family 4	Nuclear	R12C			D2D		R95R
Family 5	Lamellar				D2D		R95R
Family 6	Lamellar						Y17Y, R95R
Family 7	Nuclear				D2D		Y17Y, R95R
Family 8	Nuclear+Microcornea				D2D		Y17Y, R95R
Family 9	Nuclear				D2D		
Family 10	Nuclear			Y56X	D2D		Y17Y, R95R
Family 11	Lamellar				D2D		Y17Y

The degree of opacification of lamellar cataracts showed some variability among families. Families 6 and 11 presented with lamellar cataracts significantly less dense than those in Families 2 and 5.

Mutations were observed in two families (18.2%). In nine families, no mutation in *CRYAA*, *CRYGC*, and *CRYGD* was detected. The cataract phenotypes in families without mutations were bilateral nuclear cataract since birth in affected members from Families 1, 3, 7, 8,  and 9 and bilateral lamellar in affected members from Families 2, 5, 6, and 11.

The proband of Family 8 who is of consanguineous origin presented with nuclear cataract and microcornea like the affected father and mother. The father had retinal detachment in both eyes after cataract surgery and the mother postoperative glaucoma. In Family 1, all affected members also had nuclear opacity and microcornea. This family comprised of 8 affected and 24 unaffected members spanning four generations. The visual acuity of the proband was 20/40 in both eyes after cataract surgery. Nystagmus was present in some families and absent in others, depending primarily on the degree of visual impairment during the first months of life.

### Mutations in *CRYAA*, *CRYGC*, and *CRYGD* associated with human cataract

In 2 of the 11 probands (from Families 4 and 10), unique mutations in one of the three genes were identified cosegregating in a heterozygous condition with the disease in the each family. In both families, the congenital bilateral nuclear cataract was present in all affected individuals.

**Figure 1:**
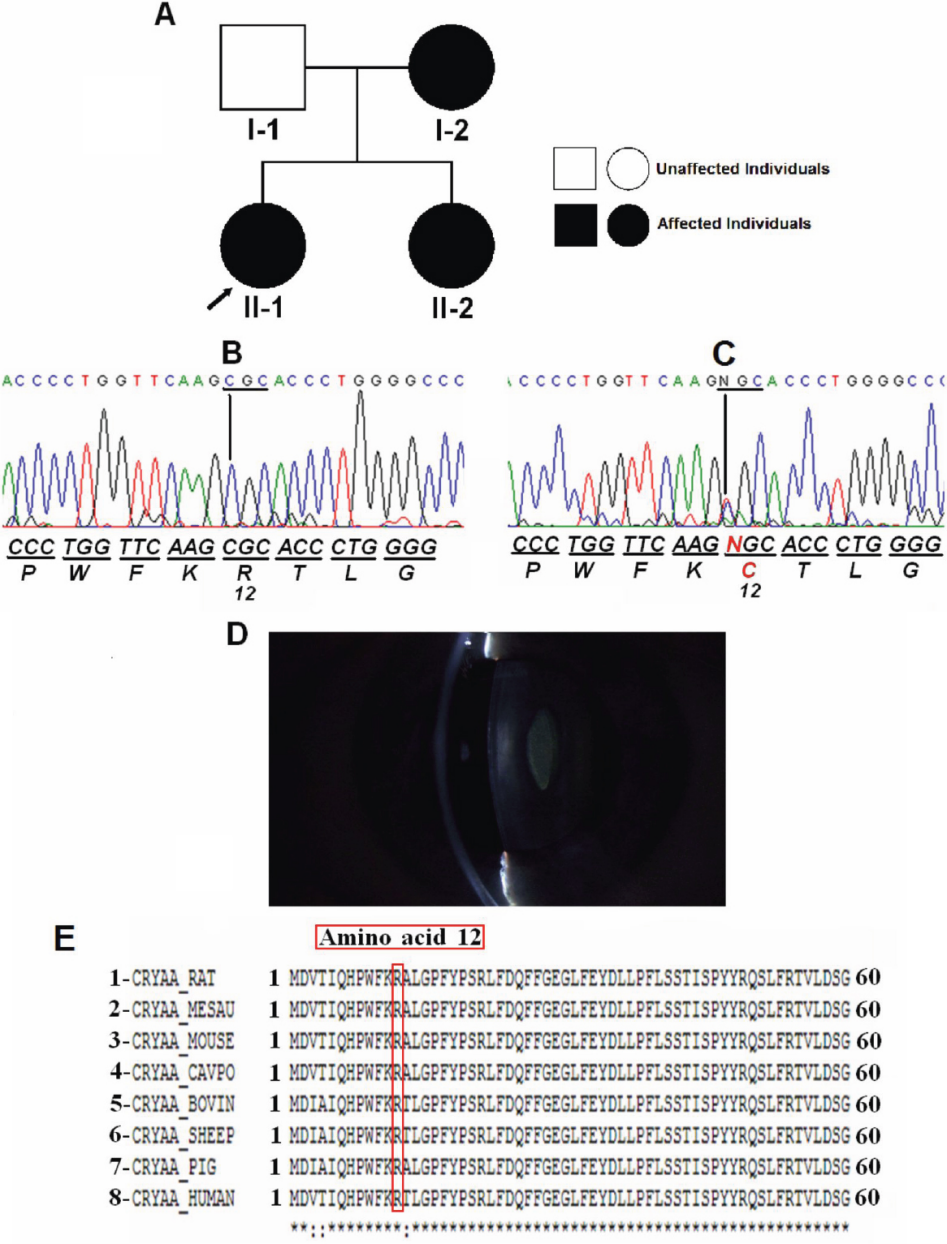
Mutation analysis of *CRYAA* in Family 4. **A:** Pedigree of Family 4 shows the proband, which is indicated by the arrow. **B:** Direct sequencing of the PCR product encompasses exon 1 of *CRYAA* (5′→3′) of an unaffected individual (I-1). **C:** Direct sequencing of the PCR product encompassing exon 1 of *CRYAA* of an affected individual (II-1) shows a heterozygous C→T transition that replaced arginine by cysteine at amino acid 12 (R12C). The mutated sequence is shown in red. **D:** The slit-lamp photograph of individual I-2 shows a nuclear cataract. **E:** Alignment of residues 1–60 of human (8) αA-crystallin protein with rat (1), hamster (2), mouse (3), guinea pig (4), cow (5), sheep (6), pig (7) is shown. The R12 residue is marked in red.

Family 4 comprised of two generations with three affected members and one unaffected member (Figure 1). Visual acuity of the affected eyes ranged from 20/80 to counting fingers after cataract surgery. In all cases, there was amblyopia. The congenital bilateral nuclear cataract in this family is associated with a mutation in *CRYAA*, a point mutation in exon 1 (**C**GC>**T**GC), which leads to the replacement of an arginine at position 12 for a cysteine (R12C). This mutation was observed in all affected members (I-2, II-1, and II-2) and was not observed in the unaffected member (I-1). There was no evidence of other ocular or systemic abnormalities. This substitution resulted in the loss of a HhaI restriction site. Wild type control PCR products were digested into fragments of 286 bp, 96 bp, and 84 bp while the presence of the R12C mutation resulted in an undigested fragment of 382 bp as well as a common 84 bp fragment. Restriction digestion of 50 normal controls did not detect the mutation.

Computational program analysis of the R12C mutation revealed the following results: in the PolyPhen the score was 2.664, which means with high confidence that this variant is predicted to be “probably damaging.” Align-GVGD showed a score of 179.53 when “deleterious” mutations tend to have scores greater than 100. Finally, the SIFT method revealed a score of 0.00 to position R12. Positions with a probability less than 0.05 are predicted to be intolerant.

**Figure 2:**
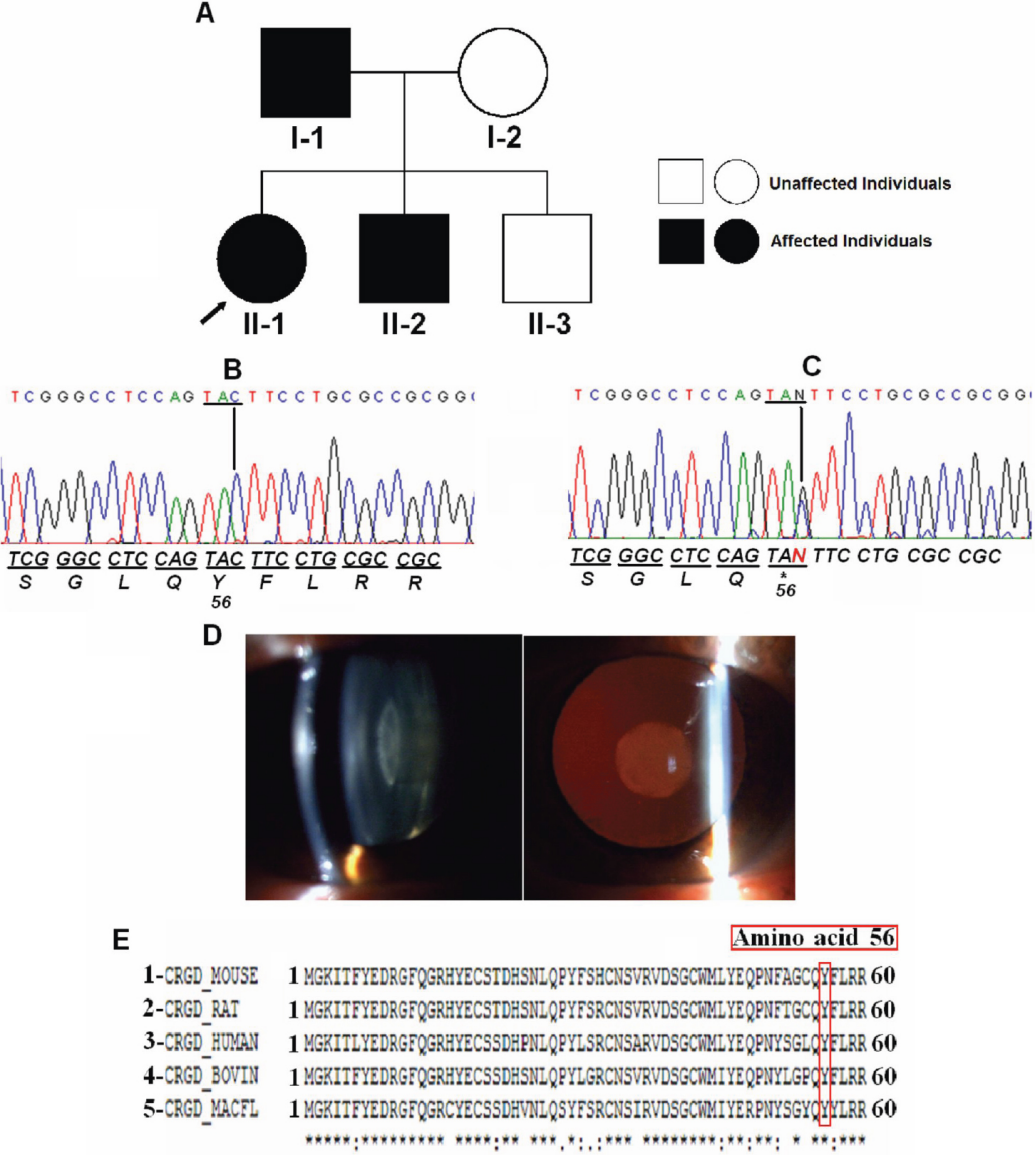
Mutation analysis of *CRYGD* in Family 10. **A:** Pedigree of Family 10 shows the proband, which is indicated by the arrow. **B:** Direct sequencing of the PCR product encompasses exon 2 of *CRYGD* of an unaffected individual (I-2). **C:** Direct sequencing of the PCR product encompassing exon 2 of *CRYGD* shows a heterozygous TAC>TAG transition that replaced a tyrosine by a premature stop codon at amino acid 56 (Y56X) in individual II-1. **D:** The photograph of the anterior eye with lens image of individual II-1 shows nuclear cataract. **E:** Multiple alignment of amino acid sequence of γD-crystallin protein with different species is shown: mouse (1), rat (2), human (3), cow (4), and kangaroo (5). The Y56 residue is marked in red.

The proband of Family 10 was diagnosed with congenital nuclear cataract. DNA sequencing analysis of *CRYGD* showed a novel heterozygous nonsense mutation (TA**C**>TA**G**) within the second exon (Figure 2). Cataract was most likely caused by this point mutation that leads to the replacement of a tyrosine by a premature stop codon at position 56 (Y56X).This mutation was observed in the proband (II-1), her affected father (I-1), and affected brother (II-2). The unaffected mother and the unaffected other brother did not show this mutation. On clinical examination, the affected members achieved a visual acuity of 20/40 in both eyes (I-1, II-2) as well as 20/50 and counting fingers in the right eye and left eye, respectively, of the proband. No other ocular findings were observed. This transition resulted in the loss of an RsaI restriction site, generating fragments of 396 bp, 108 bp, and 92 bp for the wild type allele and fragments of 488 bp, 396 bp, 108 bp, and 92 bp for the mutant allele. The analysis of 50 normal controls did not detect the substitution.

### Polymorphisms in *CRYAA*, *CRYGC*, and *CRYGD* 

A variety of sequence variations referred as single nucleotide polymorphisms (SNPs) was observed in the probands for *CRYAA*, *CRYGC*, and *CRYGD*. None of these sequence changes in the coding regions led to amino acid alterations. Three known polymorphisms [34-36] were observed in the sequencing analysis, D2D (rs872331) polymorphism (GA**C**>GA**T**) in *CRYAA* (exon 1), which was observed in 10 probands; Y17Y (rs2242074) polymorphism (TA**T**>TA**C**) in *CRYGD* (exon 2), which was observed in seven probands; and R95R (rs2305430) polymorphism (AG**A**>AG**G**) in *CRYGD* (exon 3), which was observed in nine probands. A new polymorphism in the third exon of *CRYGC* (S119S) was observed in Family 1. This alteration (AG**C**>AG**T**) resulted in the gain of a novel MslI restriction site, producing fragments of 387 bp and 203 bp while the wild type allele showed a fragment of 590 bp. Fourteen members of the family (seven affected members and seven unaffected members) were analyzed by restriction enzyme digestion. This polymorphism was observed in the heterozygous state in five affected members, and it was not detected in the unaffected individuals. Digestion analysis of 50 healthy unrelated individuals also did not detect the substitution.

## Discussion

The transparency and high refractive index of the lens are achieved by the precise architecture of the fiber cells and the homeostasis of the lens proteins in terms of their concentration, stability, and supramolecular organization [6]. Pras et al. [37] estimated that approximately 30 loci are involved in autosomal dominant human congenital cataract. Different crystallin genes have been recognized as the main candidates for certain hereditary forms of lens opacity in humans [12].

In this study, we identified a mutation in the first exon of *CRYAA* (R12C) in Family 4. The affected family members presented with nuclear cataract and had no other ocular defects. Since this mutation segregates with the disease within this family and could not be detected in 50 controls, we consider this allele as the probable causative molecular lesion for the observed clinical findings. The abnormal protein produced for this heterozygous mutation may precipitate in the lens and therefore induce the precipitation of other proteins [37]. During the course of this study, the same R12C substitution was independently associated with congenital central, zonular cataract with microcornea in a Danish family [21]. The difference between the phenotype reported in this Danish family and that observed in our Brazilian family may be related to the effect of an unknown modifier gene or to sequence variation within the regulatory region that could affect the expression of *CRYAA*. Previous studies have already reported phenotypic heterogeneity of the disease with the same mutation in *CRYAA* [38].

The αA-crystallins belong to the small heat shock protein family and function as chaperones. They all share a common structure of an NH_2_-terminal less-conserved region, a conserved α-crystallin domain, and a short COOH-terminal. Molecular chaperones facilitate the correct folding of proteins in vivo and are of extreme importance in keeping these proteins properly folded and in a functional state [39].

The R12C mutation is located in the NH_2_-terminal portion, and the alignment of the primary sequence for small heat shock proteins from plants, bacteria, and mammals, including human CRYAA, demonstrate that the arginine residue is highly conserved at this position. The conserved αA-crystallin domain region may be involved in aggregation and disaggregation of larger protein complexes whereas the NH_2_-terminal and the COOH-terminal regions might play a role in oligomerization [40]. Astonishingly, all dominant mutations reported in *CRYAA* have the basic amino acid arginine involved. Thus, this residue probably has a very important role in maintaining the structural integrity of the lens [41].

We evaluated the likely effect of amino acid substitution (R12C) on αA-crystallin protein function using PolyPhen, SIFT, and Align-GVGD computational programs. Chan and colleagues [32] demonstrated that the isolated predictive value of these programs can be increased by their combination. These programs predict the possible deleterious effect of an amino acid variant on the structure and function of a protein based on sequence homology and structural information. Significantly, all three different algorithms considered the R12C mutation as possibly damaging to the αA-crystallin protein.

Molecular analysis of *CRYGD* of the affected members of Family 10 showed a novel heterozygous nonsense mutation in exon 2 (TA**C**>TA**G**). This mutation resulted in the substitution of the tyrosine residue 56 by a premature stop codon (Y56X), resulting in a truncated protein missing 118 amino acids.

An increasing number of mutations in *CRYGD* have been described in association with human congenital cataract [41]. Hansen et al. [21] related that the mechanisms through which protein abnormalities cause loss of lens transparency are still speculative. These truncated protein products may act by a dominant negative mechanism, giving rise to the cataract phenotypes in this family. The γ-crystallin proteins are a superfamily of proteins that have a Greek Key (GK) motif unit base [28]. They contain two domains, an NH_2_-terminal domain and a COOH-terminal domain as the core. Each domain contains two GK motifs. Each GK motif is composed of four antiparallel β-strands. The two domains are connected by a distinct connecting peptide [19]. Based on the crystal structure of human γD-crystallin, the Y56X substitution resulted in a complete absence of the COOH-terminal domain, leading to a major change in structural conformation of the protein. This hypothesis is further supported by the fact that γD-crystallins have shown a strong tendency to maintain conservation throughout evolution in different species. The computational programs used in this study are not able to predict the impact of nonsense mutations on protein function. They only apply to missense mutations.

A new polymorphism in the third exon of *CRYGC* (S119S) was observed in Family 1. It was present in five of the seven affected individuals and absent in all unaffected individuals as well as in the control group. We might consider that it represents a rare polymorphism or that it may be exclusive of this sample of Brazilian patients. Another hypothesis is that this polymorphism could be a marker of an unidentified gene located in this region and that its absence in two of the affected individuals would be due to recombination events. This fact would make this four-generation family a target to the analysis of microsatellite markers surrounding the location of the polymorphism.

In conclusion, we report a novel nonsense mutation (Y56X) in *CRYGD* and a previously reported missense mutation (R12C) in *CRYAA* associated with nuclear cataract in Brazilian families. Additionally, we also observed a new polymorphism (S119S) in *CRYGC*. The analysis of the remaining nine families reported here excluded possible mutations in *CRYAA*, *CRYGC*, and *CRYGD*, suggesting that other genes/loci could be involved with congenital nuclear/lamellar cataract.
